# Venomics and antivenomics of the poorly studied Brazil’s lancehead, *Bothrops brazili* (Hoge, 1954), from the Brazilian State of Pará

**DOI:** 10.1590/1678-9199-JVATITD-2019-0103

**Published:** 2020-04-17

**Authors:** Libia Sanz, Alicia Pérez, Sarai Quesada-Bernat, Rafaela Diniz-Sousa, Leonardo A. Calderón, Andreimar M. Soares, Juan J. Calvete, Cleópatra A. S. Caldeira

**Affiliations:** 1 Evolutionary and Translational Venomics Laboratory, Spanish National Research Council (CSIC), Valencia, Spain.; 2 Center for the Study of Biomolecules Applied to Health (CEBio), Oswaldo Cruz Foundation Rondônia, Porto Velho, RO, Brazil.; 3 Graduate Program in Experimental Biology (PGBIOEXP), Federal University of Rondônia (UNIR), Porto Velho, RO, Brazil.; 4 São Lucas University Center (UniSL), Porto Velho, RO, Brazil.; 5 Graduate Program in Biodiversity and Biotechnology, BIONORTE Network, Porto Velho, RO, Brazil.; 6 Aparício Carvalho University Center (FIMCA), Porto Velho, RO, Brazil.; 7 National Institute of Science and Technology in Epidemiology of the Western Amazônia, (INCT-EpiAmO), Porto Velho, RO, Brazil.

**Keywords:** Snake venom, Bothrops *brazili*, Venomics, Third-generation antivenomics, Brazilian antibothropic polyvalent antivenom

## Abstract

**Background:**

The Brazil’s lancehead, *Bothrops brazili*, is a poorly studied pit viper distributed in lowlands of the equatorial rainforests of southern Colombia, northeastern Peru, eastern Ecuador, southern and southeastern Venezuela, Guyana, Suriname, French Guiana, Brazil, and northern Bolivia. Few studies have been reported on toxins isolated from venom of Ecuadorian and Brazilian *B. brazili*. The aim of the present study was to elucidate the qualitative and quantitative protein composition of *B. brazili* venom from Pará (Brazil), and to carry out a comparative antivenomics assessment of the immunoreactivity of the Brazilian antibothropic pentavalent antivenom [*soro antibotrópico* (SAB) in Portuguese] against the venoms of *B. brazili* and reference species, *B. jararaca*.

**Methods:**

We have applied a quantitative snake venomics approach, including reverse-phase and two-dimensional electrophoretic decomplexation of the venom toxin arsenal, LC-ESI-MS mass profiling and peptide-centric MS/MS proteomic analysis, to unveil the overall protein composition of *B. brazili* venom from Pará (Brazil). Using third-generation antivenomics, the specific and paraspecific immunoreactivity of the Brazilian SAB against homologous (*B. jararaca*) and heterologous (*B. brazili*) venoms was investigated.

**Results:**

The venom proteome of the Brazil’s lancehead (Pará) is predominantly composed of two major and three minor acidic (19%) and two major and five minor basic (14%) phospholipase A_2_ molecules; 7-11 snake venom metalloproteinases of classes PI (21%) and PIII (6%); 10-12 serine proteinases (14%), and 1-2 L-amino acid oxidases (6%). Other toxins, including two cysteine-rich secretory proteins, one C-type lectin-like molecule, one nerve growth factor, one 5'-nucleotidase, one phosphodiesterase, one phospholipase B, and one glutaminyl cyclase molecule, represent together less than 2.7% of the venom proteome. Third generation antivenomics profile of the Brazilian pentabothropic antivenom showed paraspecific immunoreactivity against all the toxin classes of *B. brazili* venom**,** with maximal binding capacity of 132.2 mg venom/g antivenom. This figure indicates that 19% of antivenom's F(ab')_2_ antibodies bind *B. brazili* venom toxins.

**Conclusion:**

The proteomics outcome contribute to a deeper insight into the spectrum of toxins present in the venom of the Brazil’s lancehead, and rationalize the pathophysiology underlying this snake bite envenomings. The comparative qualitative and quantitative immunorecognition profile of the Brazilian pentabothropic antivenom toward the venom toxins of *B. brazili* and *B. jararaca* (the reference venom for assessing the bothropic antivenom's potency in Brazil), provides clues about the proper use of the Brazilian antibothropic polyvalent antivenom in the treatment of bites by the Brazil’s lancehead.

## Background

The genus *Bothrops* includes at least 50 species of pit vipers (Viperidae: Crotalinae) that are widely distributed throughout the Americas, from Mexico to southern Argentina, in different ecoregions, from tropical and subtropical forests to arid and semiarid regions, and from sea level to altitudes of more than 3000 m [1, 2]. *Bothrops* species exhibit extreme diverse morphological and ecological traits, including terrestrial, arboreal and semiarboreal species, many of which show generalist, while others show specialized dietary habits (e.g. rodents or birds), and ontogenetic shifts in diet [3]. Although still subject to taxonomic instability [4], all the clades within genus *Bothrops* include species that represent the main medically important venomous snakes in their range [5-7]. The clinical presentations of patients suffering from envenomations by viperid snakes show both local tissue damage and systemic manifestations, such as hemorrhage, coagulopathies and hemodynamic instability [6, 8]. 

In Ecuador, 1200-1400 cases of snakebites are yearly reported in 19 of the 21 provinces. East of the Andes, the principal venomous species are the common lancehead (*B. atrox*) and two-striped forest pitviper (*B. bilineatus smaragdinus*) [9]. The main clinical effects of envenomings by *B. atrox* are life threatening bleeding and blood coagulation disorders, shock, and renal failure. Other species such as *B. brazili* and *L. muta*, although potentially as dangerous as *B. atrox*, rarely bite people and envenoming by *B. b. smaragdinus* is usually less severe [9]. The vast majority of snakebites in Peru are inflicted by species of the genus *Bothrops* [10]. *Bothrops brazili*, distributed in the tropical rainforests in the eastern part of the country, is one of the main species responsible for snakebite accidents in Peru, and its venom composes the antigenic pool used to produce bothropic antivenom in this country. Peruvian bothropic antivenom (P-BAV) is an IgG solution obtained from horses immunized with a pool of venoms, consisted of 50% of *B. atrox* venom and 12.5% of pooled venom from other species (*B. pictus*, *B. barnetti*, *B. brazili* and *Bothrocophias hyoprora*) [11]. In French Guiana, *B. atrox*, *B. brazili*, *B. bilineatus*, *L. muta* and *Micrurus* sp. are responsible for most cases of snakebite envenomation [8]. Different from other Brazilian regions, *B. atrox*, *B. brazili* and *B. taeniata* are responsible for almost 90% of human accidents in the Rio Negro Amazonian region [12, 13].

Named in honor of the Brazilian physician and herpetologist Vital Brazil Mineiro da Campanha [14], founder and former director of the Butantan Institute in São Paulo, the Brazil’s lancehead, *Bothrops brazili* (Hoge, 1954) [15], is a stoutly built terrestrial venomous pit viper endemic to South America. Phylogenetic studies recover *B. brazili* and *B. jararacussu* within the “jararacussu” group, a sister branch of the monophyletic “*asper-atrox*” species clade [2, 16]. Despite being a wide-ranging species, which inhabits in lowlands of the equatorial rainforests of southern Colombia, northeastern Peru, eastern Ecuador, southern and southeastern Venezuela, Guyana, Suriname, French Guiana, Brazil (Acre, Amazonas, Mato Grosso, Maranhão, Pará and Rondônia), and northern Bolivia [1, 17, 18], no subspecies are currently recognized for the Brazil’s lancehead. Terrestrial and mainly a nocturnal snake, adults of *B. brazili* are usually 70-90 cm in total length (including tail), but may exceed 140 cm. Among adult specimens, females are much larger than males [1]. Data from specimens from the Brazilian states Maranhão, Pará and Rondônia [3], and from the upper Amazon basin, Iquitos Region, Peru [19], indicated that Brazil’s lanceheads exhibit ontogenetic shift in prey type diet from invertebrate ectotherms to vertebrate ecto- and endotherms. Centipedes are common prey items of juveniles whereas adults are generalists feeding mainly on rodents, anurans, and lizards. 

Peruvian *B. brazili* produces large amounts of venom (3-4 mL) [20] with potent median lethal dose (LD_50_) in mice of 15.27 µg/18-20 g mouse compared to 49.90 µg/mouse (*B. atrox*), 45.22 µg/mouse (*B. bilineatus*), and 58.91 µg/mouse (*B. pictus*) [11]. In the murine model, Peruvian *B. brazili* exhibited minimum hemorrhagic dose (MHD) of 7.40 µg/mouse), minimum dermonecrotic dose (MND) of 152.15 µg/mouse, minimum coagulant dose against plasma (MCD-P) and fibrinogen (MCD-F) of 19.20 and 1020.0 µg/mL, respectively, and minimum defibrinogenating dose (MDD) of 7.0 µg/mouse [11]. Although described as a new *Bothrops* from Brazil 65 years ago [15], very few studies have been reported on the toxin arsenal of the Brazil’s lancehead venom, and these were mainly focused on the pharmacological effects and possible biotechnological applications of isolated toxins [21-31], including acidic and basic phospholipase A_2_ (PLA_2_) molecules (myotoxic Braziliase I and II, MTX I and II, brazilitoxins II and III) [23-26]; a PI-snake venom metaloproteinase (SVMP), with *in vitro* antiplasmodial properties [27]; coagulant thrombin-like and pro-angiogenic snake venom serine proteinase (SVSP) [28, 29]; and a hyaluronidase [30]. 

Recently, Gren and et al. [31] reported the presence of 5′-nucleotidase (5'-NT), C-type lectin-like (CTL), L-amino acid oxidase (LAO), phosphodiesterase (PDE), phospholipases A_2_ (PLA_2_) and B (PLB), and SVMP molecules in the high molecular size-exclusion chromatographic fraction of a number of bothropic venoms, including *B. brazili* [31]. However, venoms comprise mixtures of toxins, which act jointly dysregulating receptors involved in maintaining vital systems and wreak havoc on internal organs of the prey. Understanding such integrated complex phenotype demands a holistic view of the system. With this in mind, we have applied a snake venomics approach to elucidate the qualitative and quantitative protein composition of *B. brazili* venom from Pará (Brazil), and a comparative antivenomics assessment of the immunoreactivity of the Brazilian antibothropic pentavalent antivenom against the venoms of *B. brazili* and *B. jararaca*, the latter used as a reference venom. 

## Materials and Methods

### Venom and antivenom

Pooled venom from *B. brazili* (State of Pará, Brazil) was acquired from Serpentário Proteínas Bioativas Ltda, Batatais, SP, and kept refrigerated (8°C) in the Bank of Amazon Venoms at the Center of Biomolecules Studies Applied to Health, CEBio-UNIR-FIOCRUZ-RO (register CGEN A4D12CB and IBAMA/SISBIO 64385-1). The antibothropic pentavalent antivenom (*soro antibotrópico pentavalente*, SAB; batch 1305077; production date: 05/2013) from Butantan Institute (São Paulo, Brazil) was raised in horses by conventional immunization schedules against a pool of venoms from *B. jararaca* (50%), *B. jararacussu* (12.5%), *B. moojeni* (12.5%), *B. alternatus* (12.5%) and *B. neuwiedi* (12.5%). The final formulation consists of purified F(ab')_2_ fragments generated by digestion with pepsin of ammonium sulfate-precipitated IgG molecules [32, 33]. A vial of SAB [10 mL, 29.2 mg F(ab')_2_/mL] neutralizes 50 mg of *B. jararaca* venom (the reference venom for assessing the bothropic antivenom potency in Brazil).

### **Isolation and initial characterization of *B. brazili* (Pará) venom proteins**

Crude lyophilized venom was dissolved in 0.05% trifluoroacetic acid (TFA) and 5% acetonitrile (ACN) to a final concentration of 15 mg/mL. Insoluble material was removed by centrifugation in an Eppendorf centrifuge at 13,000x*g* for 10 min at room temperature, and the proteins contained in 40µL (600 µg) were separated by RP-HPLC using a Agilent LC 1100 High Pressure Gradient System equipped with a Teknokroma Europa C18 (25 cm x 5 mm, 5µm particle size, 300 Å pore size) column and a DAD detector. The column was developed at a flow rate of 1.0 mL/min with a linear gradient of 0.1% TFA in MilliQ^®^ water (solution A) and 0.1% TFA in acetonitrile (solution B), isocratic (5% B) for 5 min, followed by 5-25% B for 10 min, 25-45% B for 60 min, and 45-70% B for 10 min. Protein detection was carried out at 215 nm with a reference wavelength of 400 nm. Fractions were collected manually across the entire elution range, dried in a vacuum centrifuge (Savant™, ThermoFisher Scientific), and redissolved in MilliQ^®^ water. Molecular masses of the purified proteins were estimated by non-reduced and reduced Tris-Tricine SDS-PAGE (on 15% polyacrylamide gels) [34], or determined by electrospray ionization (ESI) mass spectrometry (MS).

For SDS-PAGE analysis sample aliquots were mixed with ¼ volume of 4x sample buffer (0.25M Tris-HCl pH 6.8, 8% SDS, 30% glycerol, 0.02% bromophenol blue, with or without 10% 2-mercaptoethanol) and heated at 85ºC for 15 min, run under reducing conditions, and the gels were stained with Coomassie Brilliant Blue G-250. For ESI-MS mass profiling, the proteins eluted in the different RP-HPLC fractions were separated by nano-Acquity UltraPerformance LC^®^ (UPLC^®^) using BEH130 C18 (100µm x 100 mm, 1.7µm particle size) column in-line with a Waters SYNAPT G2 High Definition Mass Spectrometry System. The flow rate was set to 0.6µL/min and the column was developed with a linear gradient of 0.1% formic acid in water (solution A) and 0.1% formic acid in ACN (solution B), isocratically 1% B for 1 min, followed by 1-12% B for 1 min, 12-40% B for 15 min, 40-85% B for 2 min. Monoisotopic and isotope-averaged molecular masses were calculated by manually deconvolution of the isotope-resolved multiply-charged MS1 mass spectra. 

### Two-dimensional (IEF/SDS-PAGE) gel electrophoresis

Two-dimensional gel electrophoresis (2-DE) was performed essentially according to the manufacturer’s (GE Healthcare Amersham Biosciences) instructions unless otherwise indicated. For the first dimension, isoelectric focusing (IEF), ~150 µg of venom were dissolved in 7M urea, 2M thiourea, 4% CHAPS, and 0.5% IPG buffer pH 3-10 and applied onto 7-cm pH 3-10 nonlinear, immobilized pH gradient (IPG) ReadyStrip™ strips. IEF was carried out with an Ettan-IPGphor isoelectric focusing unit at 20°C applying the following conditions: 300 V (0.5 h), ramping to 1000 V (0.5 h), ramping to 5000 (1.3 h) and 5000 V (0.5 h). After IEF, the IPG strips were kept at -70°C until use. For the second dimension, SDS-polyacrylamide gel electrophoresis (SDS-PAGE), the IPGs were equilibrated for 15 min with gentle shaking and at room temperature in equilibration buffer [6 M urea, 2% (w/v) SDS, 30% (v/v) glycerol, 75 mM Tris-HCl, pH 8.8], with or without 40 mM DTT. IPG strips were then placed on top of SDS-15% polyacrylamide gels and run in a Protean II (Bio-Rad) electrophoresis unit at room temperature. Protein spots were visualized by Coomassie Brilliant Blue G250 staining. 

### Characterization and relative quantification of RP-HPLC fractions and 2-DE protein spots of the Brazil’s lancehead venom peptidome and proteome

Protein bands of interest were excised from Coomassie Brilliant Blue-stained SDS-PAGE and 2-DE gels and subject to in-gel disulfide bond reduction (10 mM dithiothreitol, 30 min at 65 ºC) and cysteine alkylation (50 mM iodoacetamide, 2h in the dark at room temperature), followed by overnight digestion with sequencing-grade trypsin (66 ng/µL in 25 mM ammonium bicarbonate, 10% ACN; 0.25 µg/sample), using a Genomics Solution ProGest™ Protein Digestion Workstation. Tryptic digests were dried in a vacuum centrifuge (SPD SpeedVac^®^, ThermoSavant), redissolved in 14µL of 5% ACN containing 0.1% formic acid, and 7µL submitted to LC-MS/MS. Tryptic peptides were separated by nano-Acquity UltraPerformance LC^®^ (UPLC^®^) as above. 

Doubly and triply charged ions were selected for CID-MS/MS. Fragmentation spectra were interpreted i) manually (*de novo* sequencing), ii) using the on-line form of the MASCOT Server (version 2.6) at http://www.matrixscience.com against the last update (Release 234 of October 15th, 2019) of NCBI non-redundant database, and iii) processed in Waters Corporation’s ProteinLynx Global SERVER 2013 version 2.5.2. (with Expression version 2.0). The following search parameters were used: Taxonomy: bony vertebrates; Enzyme: trypsin (two missed cleavage allowed); MS/MS mass tolerance was set to ± 0.6 Da; carbamidomethyl cysteine and oxidation of methionine were selected as fixed and variable modifications, respectively. All matched MS/MS data were manually checked. Peptide sequences assigned by *de novo* MS/MS were matched to homologous proteins available in the NCBI non-redundant protein sequences database using the online BLASTP program [35] at https://blast.ncbi.nlm.nih.gov/Blast.cgi. 

The relative abundances of the chromatographic peaks obtained by reverse-phase HPLC fractionation of the whole venom were calculated as “% of total peptide bond concentration in the peak” by dividing the peak area by the total area of the chromatogram [36-38]. For chromatographic peaks containing single components (as judged by SDS-PAGE and/or MS), this figure is a good estimate of the % by weight (g/100 g) of the pure venom component [39]. When more than one venom protein was present in a reverse-phase fraction, their proportions (% of total protein band area) were estimated by densitometry of Coomassie-stained SDS-polyacrylamide gels using MetaMorph^®^ Image Analysis Software (Molecular Devices). Conversely, the relative abundances of different proteins contained in the same SDS-PAGE band were estimated based on the relative ion intensities of the three most abundant peptide ions associated with each protein by MS/MS analysis. The relative abundances of the protein families present in the venom were calculated as the ratio of the sum of the percentages of the individual proteins from the same toxin family to the total area of venom protein peaks in the reverse-phase chromatogram.

### Third-generation antivenomics

Third-generation antivenomics [40, 41] was applied to compare the immunoreactivity of the Brazilian pentabothropic antivenom (SAB) towards the venoms of *B. brazili* and *B. jararaca* from the southeastern clade population within the Brazilian Atlantic forest [42] (used as reference venom). To this end, one vial of antivenom was dialyzed against MilliQ^®^ water, lyophilized, and 150 mg of total lyophilizate weight were reconstituted in 6 mL of 0.2 M NaHCO_3_, 0.5 M NaCl, pH 8.3 (coupling buffer). The concentrations of this antivenom stock solution [21.62 mg F(ab')_2_/mL] was determined spectrophotometrically using an extinction coefficient for a 1 mg/mL concentration (ε^0.1%^) at 280 nm of 1.36 (mg/mL)^-1^ cm^-1^ [43]. 

Antivenom affinity columns were prepared in batch. To this end, 3 mL of CNBr-activated Sepharose™ 4B matrix (Ge Healthcare, Buckinghamshire, UK) packed in a ABT column (Agarose Bead Technologies, Torrejón de Ardoz, Madrid) and washed with 15x matrix volumes of cold 1 mM HCl, followed by two matrix volumes of coupling buffer to adjust the pH of the column to 8.0-9.0. CNBr-activated instead of N-hydroxysuccinimide (NHS)-activated matrix was employed because NHS released during the coupling procedure absorbs strongly at 280 nm, thus interfering with the measurement of the concentration of antibodies remaining in the supernatant of the coupling solution. One hundred thirty mg of antivenom dissolved in 6 mL of coupling buffer were incubated with 3 mL CNBr-activated matrix for 4 h at room temperature. Antivenom coupling yield, estimated measuring A_280nm_ before and after incubation with the matrix, was 95.7 mg (i.e., 31.9 mg F(ab')_2_/mL CNBr-activated Sepharose™ 4B matrix). 

After the coupling, remaining active matrix groups were blocked with 3 mL of 0.1 M Tris-HCl, pH 8.5 at room temperature for 4 h. Affinity columns, each containing 282 µL of affinity matrix containing 9 mg of immobilized SAB F(ab')_2_ molecules, were alternately washed with three matrix volumes of 0.1 M acetate containing 0.5 M NaCl, pH 4.0-5.0, and three matrix volumes of 0.1 M Tris-HCl, pH 8.5. This procedure was repeated 6 times. The columns were then equilibrated with three volumes of working buffer (PBS, 20 mM phosphate buffer, 135 mM NaCl, pH 7.4) and incubated with increasing amounts (100-3600 µg of total venom proteins) of *B. brazili* or *B. jararaca* dissolved in ½ matrix volume of PBS, and the mixtures incubated for 1 h at 25°C in an orbital shaker. 

As specificity controls, 300µL of CNBr-activated Sepharose™ 4B matrix, without (mock) or with 9 mg of immobilized control (naïve) horse IgGs, were incubated with venom and developed in parallel to the immunoaffinity columns. The non-retained eluates of columns incubated with 100-300, 600, 900, 1200, 2400, 3600 µg of venom were recovered, respectively, with 3x, 5x, 7x, 9x, 17x and 25x matrix volume of PBS, and the immunocaptured proteins were eluted, respectively, with 3x (100-300 µg) and 6x (600-3600 µg) matrix volume of 0.1M glycine-HCl, pH 2.7 buffer, and brought to neutral pH with 1M Tris-HCl, pH 9.0. The entire fractions eluted in 100-300 µg, ½ of the fractions recovered in 600 µg, ½ of the non-retained fractions and ½ of the retained fractions recovered in 900 µg, ¼ of the non-retained fractions and ½ of the retained fractions recovered in 1200 µg, ⅛ of the non-retained fractions and ¼ of the retained fractions recovered in 2400 µg and ^1^/_12_ of the non-retained fractions and ¼ of the retained fractions recovered in 3600 µg, were concentrated in a Savant SpeedVac™ vacuum centrifuge (ThermoFisher Scientific, Waltham, MA USA) to 45µL, 40µL of which were then fractionated by reverse-phase HPLC using an Agilent LC 1100 High Pressure Gradient System (Santa Clara, CA, USA) equipped with a Discovery^®^ BIO Wide Pore C18 (15 cm x 2.1 mm, 3µm particle size, 300 Å pore size) column and a DAD detector as above. 

Eluate was monitored at 215 nm with a reference wavelength of 400 nm. The fraction of non-immunocaptured molecules was estimated as the relative ratio of the chromatographic areas of the toxin recovered in the non-retained (NR) and retained (R) affinity chromatography fractions using the equation:

$$\%NRi=100-\;\left[\;\frac{Ri}{Ri+NRi}\;\times 100\;\right]$$

where Ri corresponds to the area of the same protein “i” in the chromatogram of the fraction retained and eluted from the affinity column. However, for some toxins that were poorly recovered in the column-retained fraction owing to their high binding affinity to the immobilized antivenom likely preventing their elution from the column [44], the percentage of non-immunocaptured toxin “i” (% NRtoxin“i”) was calculated as the ratio between the chromatographic areas of the same peak recovered in the non-retained fraction (NRtoxin“i”) and in a reference venom (Vtoxin“i”) containing the same amount of total protein that the parent venom sample and run under identical chromatographic conditions, using the equation:

$$\%NRtoxin“i”=\;\frac{NRtoxin“i”}{Vtoxin“i”}\;\times 100$$

The percentage of antivenom anti-toxin F(ab')_2_ molecules was calculated by dividing [(1/2 maximal amount (in µmoles) of total venom proteins bound per antivenom vial) x molecular mass (in kDa) of antibody (F(ab')_2_, 110 kDa) molecule] by the [total amount of antibody (F(ab')_2_) (in mg) per antivenom vial] [41, 45, 46]. Binding saturation was computed by extrapolation from data modelled in Excel to degree 2 polynomial functions. 

## Results and Discussion

### ESI-MS mass profiling across the reverse-phase HPLC separation of the Brazil’s lancehead venom proteome

**Figure 1.**
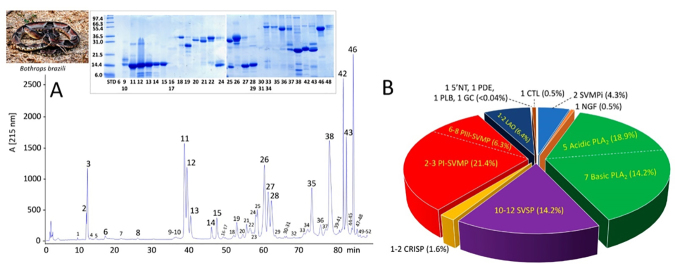
Venomics analysis of *Bothrops brazili.* **(A)** Reverse-phase chromatographic separation of the venom proteins of *Bothrops brazili* from Pará, Brazil. For venomics analysis the chromatographic fractions were collected manually and analyzed by SDS-PAGE (inset) under reduced conditions. Protein bands were excised, in-gel digested with trypsin, the resulting proteolytic peptides fragmented through LC-nESI-MS/MS, and the parent proteins identified by database searching and *de novo* sequencing followed by BLAST analysis (Additional file 1). The photograph of *Bothrops brazili* was kindly provided by Tiago Santana. **(B)** Pie chart displaying the estimated number and their relative occurrence (in percentage of total venom proteins) of toxins from the different protein families found in the venom proteome of *Bothrops brazili* (panel **A**). SVMPi: tripeptide inhibitors of snake venom metalloproteinase (SVMP); NGF: nerve growth factor; PLA_2_: phospholipase A_2_; SVSP: snake venom serine protease; CRISP: cysteine-rich secretory protein; PI- and PIII-SVMP: SVMPs of class PI and PIII, respectively; LAO: L-amino acid oxidase; 5'NT: 5'-nucleotidase; PDE: phosphodiesterase; PLB: phospholipase B; CTL: C-type lectin-like.

The venom proteome of 600 µg of crude venom of *B. brazili* (Pará) was decomplexed and quantified by reverse-phase HPLC and downstream SDS-PAGE analysis of the chromatographic peaks (Fig. 1, Additional file 1). Twenty major and 25 minor chromatographic peaks were recovered, and the electrophoretic analysis of these fractions showed that most comprised a major component and a variable number of minor bands (Fig. 1, inset). Since only proteins with identical chemical formulae are isobaric, mass profiling represents a convenient approach for identifying a venom by means of its mass fingerprint and differentiating it not only from other species' venoms but also from geographical variants within the same species [47, 48]. To highlight molecular markers of *B. brazili* (Pará) venom investigated in this work, RP-HPLC fractions 2-46 were submitted to molecular mass determination by LC-ESI-MS mass profiling. 

Chromatographic peaks 2 (m/z 430.3) and 3 (m/z 444.4), which accounted for 0.76% and 3.55% of the total RP-HPLC chromatogram area (Additional file 1) contained, respectively, the tripeptides ZNW (pyroGlu-Asn-Trp) and ZBW (pyroGlu-Lys/Gln-Trp), characterized as weak endogenous inhibitors (IC_50_ in the range of 0.15-0.95 mM) of the fibrinogenolytic activity of multiple snake venom Zn^2+^-metalloproteinases (SVMP) [49]. These peptide inhibitors regulate the proteolytic activities of SVMPs in a reversible manner under physiological conditions [50]. It is thus conceivable that they may protect glandular tissues and venom factors from the proteolytic activity of SVMPs stored at high concentration in an inactive but competent state for many months in the lumen of the venom gland of many Viperidae snakes [49, 51-53].

A number of chromatographic peaks showed fairly well isolated proteins of intact isotope-averaged molecular masses (M_ave_) in the range expected for phospholipase A_2_ (PLA_2_) molecules, 13,948,1 Da, 13,888.7 Da and 13,850.3 Da [Fr. 9-10, 0.85% by weight of the total venom components (TVC), Additional file 1]; 13,833.6 Da (Fr 11, 8.6% TVC); 13,872.5 Da (Fr 12, 7% TVC); 13,935.6 Da (Fr 13, 2.3% TVC); 13,929.7 Da (Fr 14, 0.7% TVC); 13,732.1 Da (Fr 15, 1.5% TVC); 13,914.8 Da (Fr. 24-25, 2% TVC); 13,855.9 Da (Fr 27, 5.4% TVC); and 13,786.9 Da (Fr 28, 4.5% TVC) (Fig. 1, Additional file 2). 

In addition, RP-HPLC fractions 18, 21, 22 and 26, all dominated by proteins migrating by SDS-PAGE at apparent molecular weights of 36,000, yielded ESI-MS masses [in Da] of 27,623.1, 27,455.2 and 13,930.5; 27,636.5 and 13,803.2; and 30,360.4, 29,663.4 and 13,781.9, respectively. These molecular masses may correspond to the minor (<0 .1% TVC) PLA_2_ molecules that co-eluted with the major SVSPs in the RP-HPLC separation (Fig. 1, inserted SDS-PAGE analysis). It is worth noting that none of the measured molecular masses match previously reported values recorded for conspecific PLA_2_ molecules, e.g., brazilitoxin-II (PDB 4K09) (pI 9.0, M_ave_: 13,741.1 Da); MTx-II (4K06) (pI 9.0, 13,713.1 Da) [25]; MTx-II (4DCF) (pI 8.9, 13836.0 Da) [54]; Braziliase-I (pI 5.2, M_ave_: 13,894.4); Braziliase-II (pI 5.3, 13,869.6) [26]. These proteins were purified from the venom of *B. brazili* of undisclosed geographic origin provided by Serpentário Sanmaru Ltda, Taquaral, São Paulo, Brazil [54] or Serpentário Proteínas Bioativas Ltda, Batatais, São Paulo, Brazil [26], strongly suggesting the occurrence of population-specific PLA_2_ molecules among *B. brazili* venoms. Intraspecific compositional variation between venoms among specimens inhabiting different geographic regions has long been appreciated by herpetologists and toxinologists as a general feature of highly adaptable and widely distributed snake species, such as *B. atrox* [47, 48], and may be due to evolutionary environmental pressure acting on isolated populations.

Venom proteins eluting in reverse-phase chromatographic fractions 18 (M_ave_: 29,899.2 Da, 30,130.2 Da and 30,421.9 Da), 19 (M_ave_: 24,850.5 Da), 20 (M_ave_: 28,318.0 Da), 38 (M_ave_: 23,090.5 Da) and 42/43 (M_ave_: 23,317,0 Da) (Fig. 1) were tentatively assigned to a cysteine-rich secretory protein (CRISP) (Fr. 19), SVSPs (Fr. 18 and 20), and PI-SVMPs (Fr. 38, 42 and 43).

As a whole, the above data suggested that the Brazil’s lancehead venom comprised nine minor (< 2.5% of total venom proteome) and five major (> 4.4%) PLA_2_s, which together account for approximately 30% (w/w) of its proteome, one minor (1.6%) CRISP molecule, one major (Fr. 26, 8.7%) and at least ten minor (< 2.3%) SVSPs, and 2-3 abundant (5.5-5.7%, Fr. 42 and 43) and a major (> 13%, Fr. 38) PI-SVMPs. In addition, SDS-PAGE analysis displayed in Figure 1 also indicated the presence in the venom of a number of protein bands compatible with minor (< 1.8%, Fr. 33, 34, 36 and 37) and major (5.9%, Fr. 35 and 10.3%, Fr. 46) LAO and/or PIII-SVMP molecules.

### **Bottom-up proteomic analysis of the toxin arsenal of *Bothrops brazili* venom from the Brazilian State of Pará**

**Figure 2.**
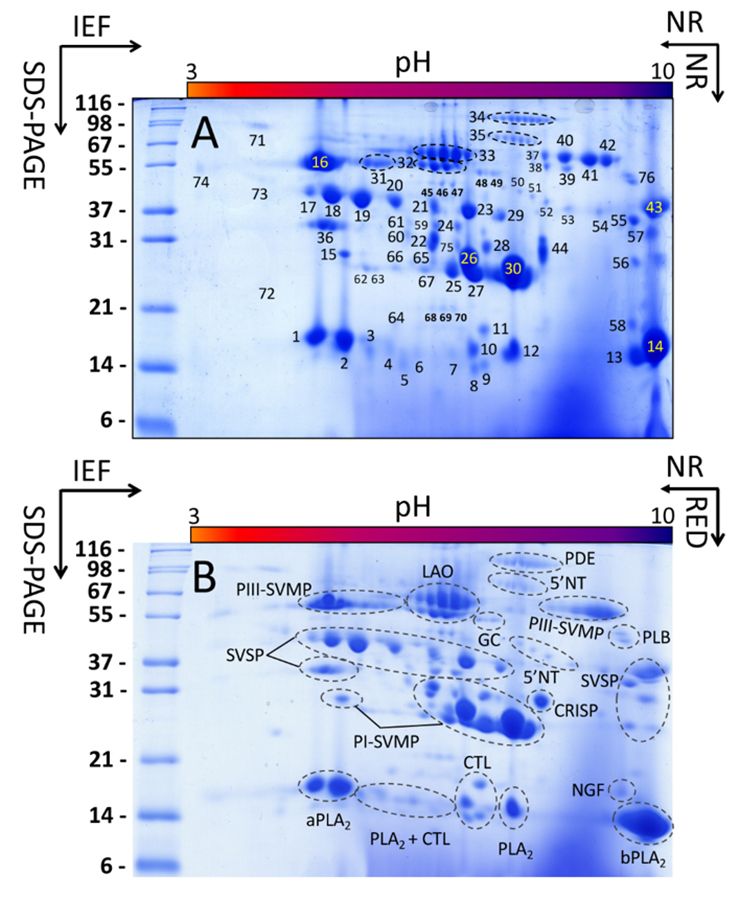
Two-dimensional gel electrophoresis of the Brazil’s lancehead venom proteome. Two-dimensional electrophoretic separations (IEF/SDS-PAGE) of the venom proteins of *Bothrops brazili* from Pará. For the first (IEF) dimension, the venom proteins were focused to their isoelectric points under non-reducing (NR) conditions. For the second (SDS-PAGE) dimension, the IPGs were equilibrated at room temperature in equilibration buffer **(A)** without (NR) or **(B)** with (RED) 40 mM of the disulfide bond reducing agent, DTT. **(A)** Spots submitted to in-gel trypsin digestion and bottom-up peptide-centric MS/MS analysis are numbered, whereas identified proteins are listed in Additional file 2. **(B)** An overview of the distribution of, and occurrence of proteoforms within, the different toxin classes identified in the venom of the Brazil’s lancehead.

Venom of the Brazil’s lancehead (Pará) was fractionated by RP-HPLC/SDS-PAGE (Fig. 1A) and 2-DE (Fig. 2). The 1D and 2D electrophoretically-resolved protein bands were submitted to in-gel trypsin digestion and bottom-up peptide-centric MS/MS analysis, followed by database matching through the online MASCOT search engine or BLAST analysis of *de novo* gathered peptide ion sequences (Additional files 1 and 2). 

Figure 1B displays the relative abundances (in percentage of the total venom proteins) of the peptide and protein classes identified. The venom proteome of *B. brazili* (Pará), comprised by at least 40-47 components (Fig. 1B), is composed predominantly by two major and three minor acidic (19%) and two major and five minor basic (14%) PLA_2_ molecules, 7-11 SVMP of classes PI (2-3, 21%) and PIII (5-8, 6%), 10-12 SVSPs (14%) and 1-2 LAOs (6%). Other toxin classes are: two CRISPs, one C-type lectin-like (CTL), one nerve growth factor (NGF), one 5'-nucleotidase (5'NT), one phosphodiesterase (PDE), one phospholipase B (PLB), and one glutaminyl cyclase (GC) represent together less than 2.7% of the venom proteome (Fig. 1B). This toxic arsenal may account for the potent median lethal dose (LD_50_) and hemorrhagic, dermonecrotic and defibrinogenating effects reported for Peruvian *B. brazili* venom in the murine model [11]. However, due to the absence of proteomic studies for that venom, any conclusion should be taken with due caution.

MS/MS analysis confirmed the lack of identity of the PLA_2_ molecules of *B. brazili* (Pará) with conspecific PLA_2_ sequences reported in the literature. PLA_2_ molecules eluted in RP-HPLC peaks 11-14 were identified as homologs of basic BrTx-II [4K09] and MTx-II [I6L8L6, 4K06, 4DCF] [25, 54], and the tryptic peptide sequences derived from PLA_2_ in RP-HPLC peak 15, 24, 25, 27 and 28 showed high similarity with homologue internal sequences of acidic PLA_2_s Braziliase-I and Braziliase-II [26]. Clearly, the extent of geographic venom variability of *B. brazili* across its wide distribution range requires detailed future studies.

### Two-dimensional electrophoretic visualization of the Brazil’s lancehead venom proteome

Two-dimensional electrophoretic (2-DE) analysis provides a rapid way to visualize the overall venom protein complexity of a snake's venom in a single image. 2-DE and RP-HPLC/SDS-PAGE are complementary approaches that combined provide a more comprehensive view of a venom proteome than each approach separately. In addition, each of these approaches serves, by itself, a specific purpose. Thus, the presence and subunit composition of covalent complexes in a venom proteome can be conveniently addressed by comparing the 2-DE protein maps resolved under non-reducing (NRed) conditions in both directions (IEF and SDS-PAGE) versus non-reducing/reducing (Red) conditions [38]. 

Figure 2 compares the 2-DE profiles resolved in the second dimension under (A) non-reducing and (B) reducing conditions. The apparent lack of differences between both 2-DE gels clearly indicated the absence of covalently bound protein complexes. On the other hand, ESI-MS/MS sequencing of 2-DE-resolved spots labeled in Figure 2 (Additional file 2) showed the occurrence of multiple proteoforms in the range of apparent molecular weights > 55,000 exhibiting roughly the same apparent molecular mass but differing in their pI, strongly suggesting the existence of glycoforms of PIII-SVMPs (spots 31, 37-39), LAO (spots 32, 33), PDE (spot 34) and 5'-nucleotidase (spot 35) with different content of terminal sialic acid in their oligosaccharide chains. 

The molecular mass range 23-42 kDa is populated with a complex pattern of SVSP, PI-SVMP, and CRISP molecules across the pH range 5-10 (Fig. 2, Additional file 2). On the other hand, and in agreement with the results of mass profiling, the 13.5-16 kDa range comprised mainly the PLA_2_ subproteome, which is made of two major acidic (pI 4.9-5.2, spots 1 and 2), two strongly basic (pI 9.5-9.8) (spots 13 and 14), and one mildly basic (pI 7.8) (spot 12), and five low abundant PLA_2_ molecules (spots 3-7) within the pI range 5.3-7.3. The latter spots also yielded CTL peptide ions, and molecules belonging to this toxin family were identified in spots 6, 9-11 (Fig. 2, Additional file 2). 2-DE venom decomplexation confirmed the assignments listed in the Additional file 1 and additionally showed the presence in the venom proteome of a very minor glutaminyl cyclase (GC) (spots 48-49, Fig. 2A and Additional file 2).

### **Antivenomics assessment of the paraspecific immunorecognition towards *B. brazili* and *B. jararaca* toxins by the pentabothropic antivenom of Butantan Institute**

In Brazil, envenomings by bothropic species are clinically treated with equine polyspecific pentabothropic (SAB) or antibothropic-lachetic F(ab')_2_ antivenoms. Queiroz et al. [55] have reported *in vitro* qualitative (Western blot) and semi-quantitative (ELISA) evidence that these antivenoms exhibited variable paraspecific immunoreactivity towards nineteen venoms of bothropic snakes, including *B. brazili* in addition to *B. alternatus*, *B. atrox*, *B. bilineatus*, *B. castelnaudi*, *B. cotiara*, *B. erythromelas*, *B. fonsecai*, *B. hyoprorus*, *B. insularis*, *B. itapetiningae*, *B. jararaca*, *B. jararacussu*, *B. leucurus*, *B. marajoensis*, *B. moojeni*, *B. neuwiedi*, *B. pirajai*, and *B. pradoi*. 

**Figure 3.**
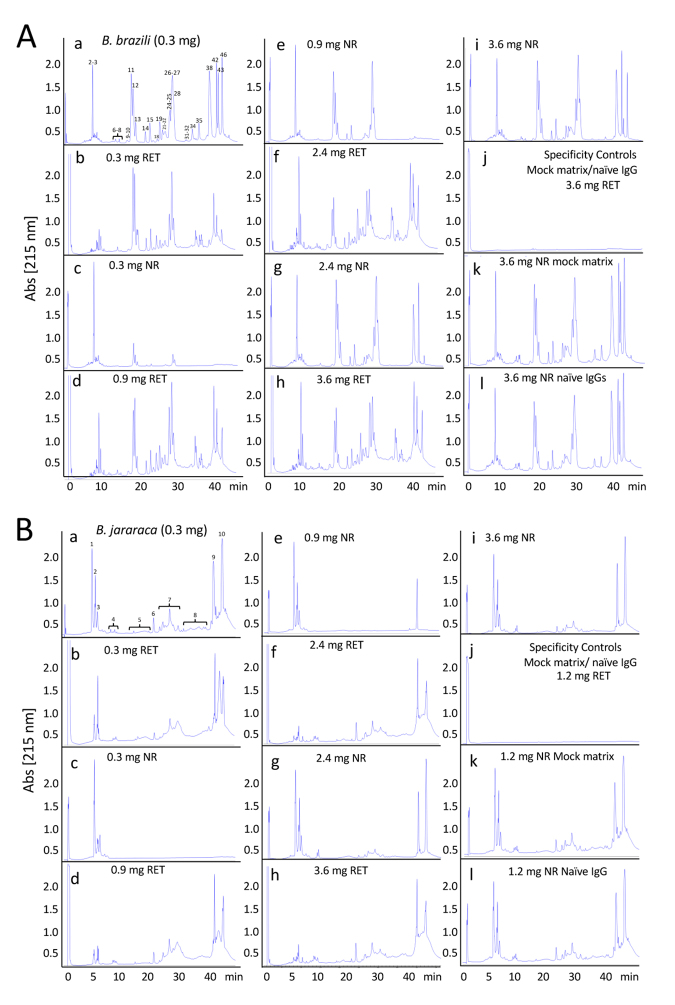
Comparative immunorecognition ability of the Brazilian SAB antivenom towards *B. brazili* and *B. jararaca* venom toxins. **(A)** Third-generation antivenomic analyses of *B. brazili* and **(B)** *B. jararaca* venom with the pentabothropic antivenom (*soro antibotrópico*, SAB) produced at Butantan Institute. Reverse-phase chromatographic analysis of whole venom (panels **a**) and of the non-retained and the immunoretained fractions recovered from affinity column [9 mg immobilized SAB antivenom F(ab’)_2_ molecules] incubated with increasing amounts (300-3600 µg) of venom from (**A**) *B. brazili* (Pará, Brazil) and (**B**) *B. jararaca* (SE population) are displayed in panels **b** through **i**. Panels **j-l** show reverse-phase HPLC separations of the retained and non-retained venom fractions on mock matrix and naïve equine IgG affinity columns, respectively.

Here, we have applied third-generation antivenomics [40, 41] to compare the qualitative and quantitative immunorecognition capability of the SAB antivenom produced at Butantan Institute (SP, Brazil) toward the venom toxins of *B. brazili* (Pará) and *B. jararaca* (reference venom). Analysis of the concentration-dependent immunocapturing profile of the SAB antivenom affinity columns showed paraspecific immunoreactivity against all the toxin classes of *B. brazili* venom (Fig. 3A, Table 1). The maximal binding capacity of immobilized (9 mg) SAB F(ab’)_2_ antibodies was 1,194.2 µg of *B. brazili* venom proteins, which correspond to 132.2 mg venom/g antivenom, or 38.6 mg of total venom proteins per vial. For a calculated average molecular mass of 35.6 kDa/venom toxin molecule, and assuming that at maximal binding both F(ab')_2_ antigen-recognition sites were occupied, the antivenomics results suggest that 19% of the SAB antibodies recognized toxins from *B. brazili* venom. This figure fall within the range of percentages (6-28%) of antitoxin antibodies determined for a number of commercial antivenoms [45; JJC, unpublished results]. 

**Table 1.** Concentration-dependent immunoretained (RET) *Bothrops brazili* (Bbr) venom proteins by SAB antivenom affinity column. Maximal binding for each RP-HPLC fraction is highlighted in bold face

***Bothrops brazili* total venom proteins ( µg)**										
RP-HPLC fraction		100	300	600	900	1200	2400	3600	Extrapolation	Toxin class
**2-3**	µg TOTAL	10.09	30.27	60.55	90.82	121.09	242.18	363.28		**SVMPi**
	µg RET	0.00	0.00	0.00	0.00	0.00	0.00	0.00		
**6-8**	µg TOTAL	1.52	4.56	9.13	13.69	18.25	36.50	54.76	76.00	**DC fragment**
	µg RET	1.52	3.18	6.34	8.87	12.03	19.56	21.69	**25.15**	
**9-10**	µg TOTAL	0.31	0.93	1.87	2.80	3.73	7.46	11.20		**PLA_2_, NGF**
	µg RET	0.31	0.93	1.87	2.80	**3.47**	2.98	2.98		
**11**	µg TOTAL	7.93	23.80	47.60	71.40	95.20	190.39	285.59	396.50	**PLA_2_**
	µg RET	7.93	19.22	21.09	16.71	20.18	21.43	22.80	**25.85**	
**12**	µg TOTAL	6.29	18.87	37.73	56.60	75.47	150.94	226.40		**PLA_2_**
	µg RET	6.29	17.01	21.99	21.90	21.06	**23.05**	22.45		
**13**	µg TOTAL	1.97	5.90	11.80	17.69	23.59	47.18	70.78		**PLA_2_**
	µg RET	1.97	5.05	6.15	6.11	6.05	**6.40**	6.38		
**14**	µg TOTAL	0.83	2.50	5.00	7.51	10.01	20.02	30.02		**PLA_2_**
	µg RET	0.83	2.30	3.37	3.84	**4.35**	3.90	3.78		
**15**	µg TOTAL	1.61	4.82	9.63	14.45	19.26	38.52	57.78		**PLA_2_**
	µg RET	1.61	4.46	6.35	**6.69**	6.62	6.16	6.03		
**16-17**	µg TOTAL	0.21	0.62	1.24	1.85	2.47	4.94	7.42	10.50	**SVSP**
	µg RET	0.21	0.62	1.24	1.85	2.47	2.94	3.06	**4.16**	
**18**	µg TOTAL	0.26	0.77	1.54	2.31	**3.08**	6.17	9.25		**SVSP**
	µg RET	0.26	0.77	1.54	2.31	**3.08**	2.68	2.34		
**19**	µg TOTAL	1.69	5.08	10.16	15.24	20.32	40.63	60.95	84.50	**CRISP**
	µg RET	1.69	5.08	10.16	15.24	20.32	23.73	25.24	**33.56**	
**20-22**	µg TOTAL	2.61	7.82	15.64	23.45	31.27	62.54	93.82	130.50	**SVSP**
	µg RET	2.61	7.25	11.81	15.04	17.78	20.35	22.73	**28.85**	
**24-25**	µg TOTAL	3.70	11.11	22.22	33.33	44.44	88.87	133.31		**PLA_2_, SVSP**
	µg RET	3.70	8.48	19.97	**25.14**	25.08	20.47	18.21		
**26-28**	µg TOTAL	15.63	46.88	93.77	140.65	187.54	375.07	562.61		**SVSP, PLA_2_**
	µg RET	15.63	45.42	**45.52**	42.40	41.17	43.65	41.43		
**30-31**	µg TOTAL	0.28	0.83	1.67	2.50	3.34	6.67	10.01		**SVSP**
	µg RET	0.28	0.83	1.67	2.50	**3.34**	3.32	3.31		
**33-34**	µg TOTAL	1.83	5.48	10.96	16.44	21.92	43.85	65.77	91.50	**SVSP**
	µg RET	1.83	5.48	10.96	16.44	21.92	22.97	33.65	**36.75**	
**35**	µg TOTAL	2.21	6.62	13.24	19.85	26.47	52.94	79.42	110.50	**LAO**
	µg RET	2.21	6.62	13.24	19.85	26.47	31.65	34.00	**43.24**	
**38**	µg TOTAL	14.60	43.79	87.59	131.38	175.18	350.35	525.53	730.00	**PI-SVMP**
	µg RET	14.17	43.24	86.01	130.62	164.29	173.27	188.76	**255.13**	
**42-43**	µg TOTAL	11.75	35.24	70.49	105.73	140.98	281.95	422.93	587.50	**PI-SVMP**
	µg RET	10.81	33.45	66.70	95.65	118.52	177.47	202.71	**235.74**	
**46**	µg TOTAL	14.70	44.10	88.19	132.29	176.39	352.78	529.16	735.00	**PIII-SVMP**
	µg RET	13.64	42.81	85.65	128.28	163.15	310.34	318.67	**384.76**	

For comparison, analysis of the concentration-dependent antivenomics profile of the SAB antivenom against the reference venom of *B. jararaca* (SE) (Fig. 3B, Table 2) showed maximal binding capacity of 1,558 µg per 9 mg F(ab')_2_ affinity column, which corresponded to 173.1 mg venom/g antivenom, or 50.6 mg of total *B. jararaca* (SE) venom proteins per vial. Assuming full occupancy of the two F(ab')_2_ antigen-recognition sites, the antivenomics results indicate that 23.7% of SAB F(ab')_2_ are toxin-binding antibodies. Moreover, the neutralization potency of the SAB antivenom specified by Butantan Institute, 50 mg of *Bothrops jararaca* reference venom/vial (10 mL), mirrors its maximal binding capacity, indicating that virtually all (50/50.6 = 98.8%) toxin-binding F(ab')_2_ antibodies may contribute to the capability of the SAB antivenom to neutralize the lethality of the homologous venom. On the other hand, the paraspecificity of SAB toward toxins of the heterologous *B. brazili* venom is due to the remarkable conservation of antigenic determinants already present in the venom of the last common ancestor of the “jararaca” and “jararacussu” clades, an event that has been dated close to the base of the radiation of genus *Bothrops* in the middle Miocene 14.07 Mya (CI95% 16.37-11.75 Mya) [56, 57].

**Table 2.** Concentration-dependent immunoretained (RET) *Bothrops jararaca* (SE) (Bj) venom proteins by SAB antivenom affinity column. Maximal binding for each RP-HPLC fraction is highlighted in bold face

***Bothrops jararaca* total venom proteins ( µg)**										
RP-HPLC fraction		100	300	600	900	1200	2400	3600	Extrapolation	Toxin class
**1**	µg TOTAL	8.62	25.86	51.71	77.57	103.43	206.86	310.28	431.00	**BPP + DISI**
	µg RET	0.45	2.50	5.77	9.18	9.17	9.94	10.96	**15.45**	
**2**	µg TOTAL	5.03	15.09	30.19	45.28	60.37	120.74	181.12	250.15	**DISI**
	µg RET	0.81	6.00	7.59	9.70	11.06	15.31	19.50	**21.15**	
**3**	µg TOTAL	2.01	6.04	12.07	18.11	24.14	48.29	72.43		**DISI + BPP**
	µg RET	0.14	0.20	0.29	0.38	0.48	**0.51**	0.51		
**4**	µg TOTAL	0.70	2.11	4.22	6.34	8.45	16.90	25.34	35.00	**DC fragment**
	µg RET	0.70	2.11	4.22	6.34	7.23	7.45	8.31	**11.21**	
**5**	µg TOTAL	2.09	6.26	12.53	18.79	25.06	50.11	75.17	104.50	**VEGF, PLA_2_**
	µg RET	2.09	6.26	9.39	9.43	9.57	10.79	16.12	**16.30**	
**6**	µg TOTAL	1.48	4.44	8.87	13.31	17.75	35.50	53.24	74.00	**CRISP**
	µg RET	1.48	4.44	8.87	12.53	16.78	30.59	34.08	**40.00**	
**7**	µg TOTAL	12.01	36.02	72.04	108.05	144.07	288.14	432.22	600.51	**SVSP, PLA_2_, CTL**
	µg RET	12.01	36.02	72.04	105.20	128.17	143.57	156.93	**206.85**	
**8**	µg TOTAL	4.03	12.10	24.19	36.29	48.38	96.77	145.15	201.50	**SVSP. CTL. LAO**
	µg RET	4.03	12.10	24.19	36.29	48.38	83.84	105.48	**119.45**	
**9**	µg TOTAL	19.20	57.61	115.22	172.84	230.45	460.90	691.34	960.01	**PI-SVMP**
	µg RET	17.92	56.07	113.23	167.29	213.21	278.24	338.59	**400.52**	
**10**	µg TOTAL	44.83	134.48	268.96	403.43	537.91	1075.82	1613.74	2191.51	**PIII-SVMP**
	µg RET	43.51	132.72	260.99	351.64	376.24	529.24	629.53	**726.54**	

### Interpretating the antivenomics outcome

Translating *in vitro* preclinical data to an *in vivo* scenario is not straightforward. Thus, although the similar total binding capacity of SAB antivenom towards *B. jararaca* and *B. brazili* venoms could be interpreted as indicative for its equivalent therapeutic potential against human envenomings by either species, the devil is in the details. In this regard, it is worth noting that although the major toxin classes PLA_2_, PIII-SVMP, PI-SVMP, and SVSP represent, respectively, 30.6%, 24.6%, 15.5%, and 13.5% of the total venom arsenal of *B. brazili*, the SAB antivenom’s antibodies contributing to its paraspecific recognition of *B. brazili* toxins are biasedly distributed against PI-SVMP (41%), PIII-SVMP (32%), SVSP (9.3%), and PLA_2_ (8.8%). This suggests that the ability of SAB to neutralize the toxic activities of Brazil’s lancehead venom associated with PIII- and PI-SVMPs, and SVSPs is equivalent to, or greater than, the *B. jararaca* reference venom. On the other hand, counteracting the toxic activities of the major *B. brazili* venom PLA_2_ molecules may require several times the amount of antivenom. 

The average venom yield of *B. brazili* is about 270 mg dry weight (biologist Luiz Henrique Anzaloni Pedrosa, Serpentário Proteínas Bioativas Ltda, Batatais, SP, Brazil, personal communication to AMS). For comparison, the average yield reported for *B. jararaca* (25-26 mg, with a maximum of 300 mg, of dry weight [58]; 40-70 mg according to the snake LD_50_ database, http://snakedatabase.org). These figures suggest that the same therapeutic potency of SAB against both venoms. However, the treatment of a Brazil's lancehead bite injecting an average amount of venom would require a 5-13 higher SAB dose than for a similar envenoming by *B. jararaca*. 

## Conclusion

The Brazil’s lancehead is a wide-ranging species endemic to lowlands of equatorial rainforests of northern South America. Phylogenetic analyses recovered two major lineages of *B. brazili* geographically restricted to regions north (Guiana Shield clade) and south (central and western Amazonian clade) of the Amazon River [59]. The divergence between these two *B. brazili* clades has been dated back to the Miocene-Pliocene border, 4.65 Mya, and the best‐fit scenario includes colonization of the Atlantic Forest from an ancestor from the Guiana Shield region through a northern bridge during the Pleistocene about 0.36 Mya, pointing to former rain forest expansions in north‐eastern South America [59]. 

Historical demographic analyses of *B. brazili* are consistent with the idea that the establishment of the Amazon River has favored divergence by promoting vicariant separation between lineages [59]. The origin of the modern Amazon River has been largely associated with the final uplift of the Andes, which led to the formation of the Amazon River, converting a widespread, northwest-flowing Miocene floodbasin into the current eastward-running Amazon Basin. The Amazon River was initiated as a transcontinental river 11.8-11.3 Mya (middle to late Miocene) and between 6.8-2.4 Mya (late Miocene to early Pleistocene) [60, 61]. The river entrenched and fully migrated onto the Amazon Fan and it was only from 2.4 Mya (late Pliocene) to the present that the Amazon fluvial system integrated regionally and acquired its current shape and size [62, 63]. These major paleogeological changes may have had major effects on the evolutionary history of the Amazonian biota. 

This work represents the first comprehensive characterization of the venom proteome of the Brazil’s lancehead. The venom was sourced from Pará, a Brazilian state south of the Amazon River. The complementary RP-HPLC/SDS-PAGE and 2-DE protein profiles of *B. brazili* venom provide a reference map for future comparative studies of the intraspecific intra- and inter-population variations of the venom proteome of this wide geographic distributed, yet poorly studied, rainforest snake species. 

The ability of SAB antivenom to recognize a broad spectrum of medically important bothropic venoms has been documented in previous works spanning the last three decades [55, 64-67]. In particular, Muniz et al. [12] reported that the Brazilian SAB antivenom neutralized the lethal activity of venoms from *B. jararaca* and *B. brazili* (obtained from a 123-cm long female collected near the high Urucu river, Coari in the Brazilian Amazonia) with potencies of 5.5 and 1.6 mg venom/mL, respectively. The antivenom showed potencies of 6.2 and 1.4 mg/mL, respectively, in the neutralization of the PLA_2_ activity of *B. jararaca* and *B. brazili* venoms. The volume of SAB antivenom that neutralized one minimal hemorrhagic dose (MHD) [68] of *B. jararaca* and *B. brazili* venoms was 5 mL and 7.8 mL, respectively. Understanding the basis of the different effectivity of SAB antivenom against homologous (*B. jararaca*) and heterologous (*B. brazili*) venoms demands the quantitative assessment of its toxin-resolved immunorecognition profile. 

Herein, we have applied third-generation antivenomics to compare the specific and paraspecific immunoreactivity of the SAB antivenom against these venoms. The remarkable paraspecificity exhibited by the Brazilian SAB antivenom against the venom of *B. brazili* is mostly due to large conservation of immunoreactive epitope on hemorrhagic PI- and PIII-SVMPs across much of the natural history of *Bothrops*. On the contrary, SAB paraspecificity against PLA_2_s, which comprise the major toxin class of the Brazil’s lancehead venom arsenal, is disproportionately diminished. Our antivenomics data allow the rationalization, in molecular terms, of the conclusions of the *in vivo* neutralization assays of Muniz et al. [12], and provide clues for designing an eventual strategy aimed at improving the spectrum of the clinical applicability of the Brazilian antibothropic polyvalent SAB antivenom.

### Abbreviations

2-DE: two-dimensional gel electrophoresis; 5'-NT: 5′-nucleotidase; ACN: acetonitrile; CTL: C-type lectin-like; GC: glutaminyl cyclase; IEF: isoelectric focusing; LAO: L-amino acid oxidase; LD_50_: median lethal dose; MCD-F: minimum coagulant dose against fibrinogen; MCD-P: minimum coagulant dose against plasma; MDD: minimum defibrinogenating dose; MHD: minimum hemorrhagic dose; MND: minimum dermonecrotic dose; NGF: nerve growth factor; NR: non-retained; P-BAV: Peruvian bothropic antivenom; PDE: phosphodiesterase; PLA_2_: phospholipase A_2_; PLB: phospholipases B; R: retained; SAB: *soro antibotrópico* (Portuguese); SDS-PAGE: SDS-polyacrylamide gel electrophoresis; SVMP: snake venom metalloproteinase; SVSP: snake venom serine proteinase; TFA: trifluoroacetic acid; TVC: total venom components. 

## Supplementary material

The following online material is available for this article:

Additional file 1. Bottom-up MS/MS identification of peptides/proteins from adult *Bothrops brazili* (Pará, Brazil) venom fractionated by RP-HPLC and SDS-PAGE as displayed in Figure 1.

Additional file 2. Bottom-up MS/MS identification of protein spots from adult *Bothrops brazili* (Pará, Brazil) venom fractionated by 2-DE as displayed in Figure 2.
